# Citrobacter freundii Methionine γ-Lyase: The Role of Serine 339 in the Catalysis of γ- and β-Elimination Reactions

**DOI:** 10.32607/actanaturae.11242

**Published:** 2022

**Authors:** N. V. Anufrieva, E. A. Morozova, S. V. Revtovich, N. P. Bazhulina, V.P. Timofeev, Ya. V. Tkachev, N.G. Faleev, A. D. Nikulin, T. V. Demidkina

**Affiliations:** Engelhardt Institute of Molecular Biology of the Russian Academy of Sciences, Moscow, 119991 Russia; Nesmeyanov Institute of Organoelement Compounds of the Russian Academy of Sciences, Moscow, 119991 Russia; Institute of Protein Research of the Russian Academy of Sciences, Pushchino, Moscow Region, 142290 Russia

**Keywords:** methionine γ-lyase, pyridoxal 5’-phosphate, Ser339, mutant form, kinetic parameters, spectral characteristics, spatial structure

## Abstract

Serine 339 of the active site of *Citrobacter freundii *methionine γ-lyase (MGL) is a conserved amino acid in most pyridoxal 5’-phosphate-dependent enzymes of the cystathionine β-lyase subclass, to which MGL belongs. The reaction mechanism of the MGL-catalyzed γ-elimination reaction is poorly explored. We replaced serine 339 with alanine using site-directed mutagenesis. The replacement of serine 339 with alanine led to a significant (by two orders of magnitude) decrease in efficiency in the catalysis of the γ- and β-elimination reactions by the mutant form of the enzyme. The exchange rates of the C-α- and C-β-protons in the amino acids in complexes consisting of the enzyme and competitive inhibitors decreased by one-two orders of magnitude. The spectral characteristics of the mutant form indicated that the replacement did not lead to significant changes in the conformation and tautomerism of MGL internal aldimine. We crystallized the holoenzyme and determined its spatial structure at 1.7 E resolution. The replacement of serine 339 with alanine did not affect the overall course of the polypeptide chain of the MGL subunit and the tetrameric enzyme structure. An analysis of the obtained kinetic and spectral data, as well as the known spatial structures of *C. freundii *MGL, indicates that serine 339 is necessary for efficient catalysis of γ- and β-elimination reactions at the stage of C-α-proton abstraction from the external aldimine, the γ-elimination reaction at the stages of coenzyme C4’-atom protonation, and C-β-proton abstraction from a ketimine intermediate.

## INTRODUCTION

Pyridoxal 5’-phosphate (PLP)-dependent enzymes underlie the vital activity of most pro- and eukaryotic organisms. These biocatalysts are characterized by the ability to perform a wide range of the chemical transformations of amino acids and amines with the involvement of the coenzyme. The substrate and reaction specificity of each particular enzyme is ensured by the interaction of the cofactor and substrate with the apoenzyme. Studies of PLPdependent enzymes and data on X-ray analysis have significantly improved our knowledge of the structure– function relationship in enzymatic catalysis and enabled, as early as 1995, the use of a rational design for changing the substrate specificity in PLPdependent catalysis [1]. Our ability to determine the crystal structures of PLP-dependent enzymes belonging to different classes has expanded our knowledge about critical residues for chemically different enzymatic reactions and engendered a number of basic ideas about biocatalysis. Replacement of the coenzyme-binding protein matrix by a matrix of the immunoglobulin superfamily has demonstrated the relevance of our main conclusions about the mechanisms of amino acid conversion by these enzymes and the possibility of predicting the changes taking place in the enzymatic activity [2 , 3, 4, 5].

Methionine γ-lyase (MGL, EC 4.4.1.11) is a PLPdependent enzyme that catalyzes the γ-elimination reaction of L-methionine, the β-elimination reaction of L-cysteine**, **and their S-substituted derivatives, as well as the γ- and β-replacement reactions of L-methionine, L-cysteine, and their analogs [6, 7]. The enzyme is found in pathogenic protozoa eukaryotes [8, 9], a number of bacteria, in particular pathogenic ones [10, 11, 12, 13, 14], fungi [15], and plants [16].

Recently, we showed that MGL catalyzes the β-elimination reaction of S-substituted cysteine sulfoxide derivatives to form thiosulfinates [17, 18]. Thiosulfinates produced by a “pharmacological pair” MGL/S-substituted L-cysteine sulfoxide exhibited antibacterial activity against a number of bacteria *in vitro* [18 , 19, 20] and *Pseudomonas aeruginosa in vivo *[21]. The applicability of the enzyme as an antitumor agent has been demonstrated *in vitro *and *in vivo *[22, 23, 24, 25].

The mechanisms of the MGL-catalyzed physiological reaction, the β-elimination reaction, and replacement reactions are poorly understood. Aside from its contribution to basic enzymology, investigation of these mechanisms is necessary for the development of new antibacterial and anticancer drugs.

MGL belongs to a cystathionine β-lyase subclass with fold type I of PLP-dependent enzymes [26]. In a tetrameric MGL molecule formed by four identical polypeptide chains, two subunits form two so-called “catalytic dimers”, each of which contains two active centers composed of amino acid residues from two subunits [27, 28].

Determination of the three-dimensional structures of the complexes between MGL and the competitive inhibitors, glycine [29], cycloserine [30], and norleucine [31, 32] has showed that the active site residue Ser339, which is conserved in the cystathionine β-lyase subclass, is most likely involved in the catalytically optimal orientation of the Lys210 side chain that binds the coenzyme.

To investigate the role of Ser339 in the catalysis of γ- and β-elimination reactions, a mutant form of the enzyme, with replacement of the serine residue with alanine (Ser339Ala MGL), was prepared by site-directed mutagenesis and the spatial holoenzyme structure, the steady- state kinetic parameters of the γ- and β-elimination reactions of several substrates, the exchange rates of the C-α- and C-β-protons in the complexes of Ser339Ala MGL with the inhibitors, and the spectral characteristics of the mutant form were determined.

## EXPERIMENTAL

**Reagents and materials**

In this study, we used L-methionine, L-norvaline, L-norleucine, L-α-aminobutyric acid, glycine, L-alanine, L-homoserine, L-homocysteine, L-phenylalanine, DL-penicillamine, phenylmethylsulfonyl fluoride, lactate dehydrogenase (LDH) from rabbit muscle, dithiothreitol (DTT), reduced nicotinamide adenine dinucleotide (NADH), and D2O (Sigma, USA); pyridoxal 5’-phosphate (Merck, Germany); S-ethyl-Lcysteine, S-methyl-L-cysteine, S-benzyl-L-cysteine, ethylenediaminetetraacetic acid (EDTA), protamine sulfate, sodium dodecyl sulfate (SDS) (Serva, USA); lactose (Panreac, Spain); glucose, glycerin, magnesium sulfate, ammonium sulfate, monobasic potassium phosphate, dibasic sodium phosphate, acetic acid, acetic anhydride, triethanolamine, HClO_4_ (Reakhim, Russia); yeast extract, tryptone (Difco, USA); DEAEcellulose (Whatmann, UK), superdex 200 (Amersham Biosciences, Sweden); Bluescript II SK(+/–) and pET28 plasmids (Novagen, USA); a DNA isolation kit and Bsp119I and BveI restriction enzymes (Fermentas, Lithuania). O-acetyl-L-homoserine was obtained by acetylation of L-homoserine [33]; D-2- hydroxyisocaproate dehydrogenase (HOHxoDH) was produced as described in [34].

**Site-directed mutagenesis**

Site-directed mutagenesis was performed using the polymerase chain reaction (PCR). The mglBlue plasmid produced by cloning the MGL gene into the Bluescript II SK(+/–) vector was used as a template in PCR. The replacement of Ser339 with alanine was performed using the following synthetic oligonucleotides (F is a forward primer, and R is a reverse primer):

(F) TATCAGCTTCGAATCGCTGGC

(RS339/A) GTATCACCGAGAGCGACCGCGA

(FS339/A) TCGCGGTCGCTCTCGGTGATAC

(R) ATACCTGCTTTAAGCCGCTCTTCTGGCGCA

After PCR, the amplicon was isolated from the reaction mixture using a DNA isolation kit. The purified DNA sample was treated with the restriction endonucleases Bsp119I and BveI and ligated with the mglBlue vector treated with the same enzymes. The resulting mixture was transformed by electroporation into *Escherichia coli *DH10B cells and grown on a solid medium (1.8% agar in Luria–Bertani (LB) medium with ampicillin). Grown colonies were transferred into a liquid LB medium supplemented with ampicillin and grown for 15–18 h. Plasmid DNA was isolated using a plasmid isolation kit and identified by analytical restriction. The fragment containing the Ser339Ala substitution was re-cloned from the mglBlue plasmid into the pET28 plasmid. The cloning accuracy was controlled by sequencing the DNA insert (Genome Center for Collective Use, Moscow). *E. coli *BL21 (DE3) cells were transformed with a plasmid containing the required insert.

**Bacterial mass cultivation and enzyme purification**

Cultivation of *E. coli *BL21 (DE3) cells containing the plasmid with the mutant gene and enzyme purification were performed as described previously [35]. The concentration of the purified enzyme was determined by absorption at 278 nm using the absorption coefficient A_1cm_^0.1%^ = 0.8 [36].

The homogeneity of samples was examined by PAGE electrophoresis under denaturing conditions [37]. During purification, activity was determined in the β-elimination reaction; activity of the final sample was determined in β- and γ-elimination reactions. The reaction mixture contained a 100-mM potassium phosphate buffer, pH 8.0, 0.1 mM PLP, 1 mM DTT, 0.2 mM NADH, 10 U LDH, and 30 mM S-ethyl-L-cysteine (the β-elimination reaction) or 70 μg HOHxoDH and 30 mM L-methionine (the γ-elimination reaction). The amount of enzyme that catalyzes the formation of 1.0 μM/min of keto acid was defined as the unit of enzymatic activity. Enzyme samples of 95% purity had a specific activity of 0.31 U/mg in the γ-elimination reaction of L-methionine and 1.03 U/mg in the β-elimination reaction of S-ethyl-L-cysteine.

**Kinetic studies**

The kinetic parameters of the γ- and β-elimination reactions were determined at 30°C in the conjugate reaction with HOHxoDH or LDH based on a decrease in NADH absorbance at 340 nm (Δε = 6,220 M^–1^cm^–1^). The reaction mixtures contained a 100-mM potassium phosphate buffer, pH 8.0, 0.1 mM PLP, 1 mM DTT, 0.2 mM NADH, 70 μg HOHxoDH or 10 U LDH, and variable amounts of the substrate in a total volume of 1 mL. The reaction was initiated by the addition of 10 μg of the enzyme.

Inhibition of the γ-elimination reaction of L-methionine by various amino acids was investigated under the conditions described above, with varying amounts of an inhibitor in the samples.

The kinetic parameters were calculated according to the Michaelis–Menten equation using the EnzFitter software [38]. In the calculations, an enzyme subunit molecular weight of 43 kDa was used. The inhibition constants were also determined using the EnzFitter software [38].

**Isotope exchange of the C-α- and C-β-protons in the enzyme complexed with the inhibitors**

The kinetics of isotope exchange reactions of the C-α- and C-β-protons for deuteron in inhibitors, catalyzed by the mutant form, was detected using 1H NMR spectroscopy. The reaction was conducted in D2O containing 50 mM potassium phosphate (pD = 7.6), 0.1 mM PLP, and an inhibitor in a total volume of 0.5 mL at 30°C at L-alanine and L-norleucine concentrations of 144.23 mM and 98.04 mM, respectively. The reaction was initiated by adding 0.3–0.7 mg of the enzyme. 1H NMR spectra were recorded at fixed time intervals on a Bruker AMXIII-400 spectrometer at an operating frequency of 400 MHz. The signals of the C-α- and C-β-protons were integrated using the modified Enzkin computer program, which is a part of the XWIN-NMR programs. The kinetic curves of the yields of deuterated products were processed according to the method described in [39].

Isotopic exchange of glycine protons was performed in D2O, pD 7.6, containing 50 mM potassium phosphate, 0.1 mM PLP, 60 mg glycine, and 3.6 mg of the enzyme. After incubation at 30°C for 72 h, the enzyme was inactivated by heating (90°C, 5 min) and separated by centrifugation. The solvent was removed on a rotary evaporator. To determine the configuration at the Cα-atom, the deuterated product was converted into a dipeptide, L-phenylalanyl-[D]-glycine, in the reaction with Boc-L-Phe-ONp [40, 41]. The dipeptide was dissolved in 0.5 mL of D2O, and the 1H NMR spectrum was collected on a Bruker AMX III-400 spectrometer at an operating frequency of 400 MHz.

**Spectral studies**

The absorption spectra of the holoenzyme in a complex with methionine were acquired at 25°C on a Cary-50 spectrophotometer (Varian, USA) in a 50-mM potassium phosphate buffer, pH 8.0, containing 1 mM DTT, 1 mM EDTA, and 20 mM L-methionine at an enzyme concentration of 1 mg/mL.

**Preparation of the holoenzyme and apoenzyme**

The apoenzyme and holoenzyme were prepared in a 50-mM potassium phosphate buffer, pH 8.0, containing 1 mM DTT and 1 mM EDTA. The holoenzyme was prepared by adding a 50-fold molar excess of PLP. The mixture was incubated at 25°C for 1 h. The excess PLP was removed by dialysis. The apoenzyme was prepared by adding a 100-fold molar excess of DL-penicillamine to the sample [42]. The mixture was incubated at 25°C for 1 h. DL-Penicillamine was removed by dialysis. The procedure was repeated until the enzyme activity decreased to 1% of the initial activity. The PLP content in the sample was determined in 100 mM NaOH using the molar extinction coefficient of PLP at 390 nm (ε = 6,600 M^–1^cm^–1^ [43]).

**Determination of the coenzyme dissociation constant**

The PLP dissociation constant was determined by ultrafiltration [44]. Varying amounts of PLP (in a range of 5 × 10^–3^ to 1.2 × 10^–1^ mM) were added to the 8 × 10^–3^ mM apoenzyme in a 50-mM potassium phosphate buffer, pH 8.0, containing 1 mM DTT and 1 mM EDTA. After incubation at 30°C for 30 min, free PLP was separated by centrifugation in a Centricon-30 microconcentrator (Amicon, United States) at 5,000 rpm and 4°C for 5 min. The PLP content in each sample was determined in 100 mM NaOH. The data were processed in Scatchard coordinates [45].

**Crystallization and data collection**

Ser339Ala MGL crystals were produced by vapor diffusion in a hanging drop under the conditions presented in [28]. Crystals suitable for the acquisition of diffraction data formed within 10 days and had a rhombic shape. Diffraction data were recorded on a synchrotron radiation source at BESSY BEAMLINE 14.1 (Berlin, Germany) using a MARMOSAIC 225 mm CCD detector at 100 K and processed in the XDS software package [46] (*Table 1*).

**Table 1:** X-ray data collection and refinement statistics of Ser339Ala MGL

Space group	I222
Unit cell parameters, Å	a = 56.66, b = 123.09, c = 128.79, α = β = γ = 90°
Wavelength, Å	0.91841
Resolution, Å	30.0–1.70 (1.79–1.70)
Completeness, %	99.4 (97.5)
Redundancy	7.0 (6.3)
R_merge_, %	4.5 (34.9)
Disordered amino acid residues in the protein	1, 46–61, 398
Number of non-hydrogen atoms in the protein	2,879
Number of water molecules	393
Number of unique reflections	49,574 (7,001)
R/R_free_	0.173/0.205 (0.243/0.285)
Average temperature B-factor, Å solvent macromolecule	29.28 27.51 41.64
Root mean deviation	
bond lengths	0.007 Å
bond angles	1.095°
chiral angles	0.047°
planar angles	0.005°
Ramachandran plot, residues in	
most favorable regions, %	98.64
additional allowed regions, %	1.36
outlier region, %	0.0

Values in parentheses are for the highest resolution shell.

**Determination and refinement of the Ser339Ala MGL spatial structure**

The structure of Ser339Ala MGL was solved by molecular replacement using the CCP4 software package [47]. As a model, we used the previously determined structure of *C. freundii *MGL (1.35 Å, PDB code 2RFV) where mobile enzyme regions, water molecules, and the coenzyme were excluded. An electron density map was calculated for the produced model. To further calculate the structure, we used the Phenix refinement protocols [48] with energy minimization and optimization of the model’s geometry, followed by manual rebuilding in the COOT program [49]. To control the refinement process, we used the model reliability factor R_free_ calculated from 5% of the reflections excluded from the refinement. The final model was refined to a resolution of 1.70 Å with the R_work_ and R_free_ parameters of 17.3% and 20.5%, respectively. The model contained 2,879 non-hydrogen atoms and 393 water molecules; the Lys210 side chain was covalently linked to PLP. An excess electron density was present near the sulfur atom in three cysteines (Cys4, Cys193, and Cys245), indicating their oxidized states, after which they were replaced with 3-sulfenoalanines. The structure was deposited into the protein data bank (PDB code 5D5S); the statistics of structure refinement are shown in *Table 1*.

## RESULTS AND DISCUSSION

The mechanism of the γ- and β-elimination reactions, shown in Scheme 1, was proposed in [50] and [51]. Because of the presence of the coenzyme, PLPdependent enzymes have unique spectral properties that enable the identification of the reaction intermediates. The main absorption bands of an internal aldimine and intermediates of the γ- and β-elimination reactions are shown in *Scheme* according to [52].

In PLP-dependent enzymes catalyzing various reactions that start with C-α-proton abstraction in the external aldimine, the lysine residue that binds the coenzyme is the base accepting this proton. In the β-elimination reaction of sulfur-containing amino acids, which is catalyzed by cystathionine γ-lyase subclass enzymes, the coenzyme-binding lysine residue was supposed to be also a general acid catalyst at the stage of leaving group elimination [51, 53].

ystathionine γ-lyase was thought to transfer a proton from the Cα-atom to the C4’-atom of PLP. Given the obtained structural data, the authors concluded that the side chain of this residue “swings like a liana” to either the Cα-, C4’-, or Cβ-atoms of the substrate, and that its ε-amino group is also a base that accepts the C-β-proton.

An analysis of the spatial structure of *C. freundii* MGL complexed with glycine, which models the structure of an external aldimine, suggested that the PLP-binding Lys210 provides both the 1,3-prototropic shift of the C-α-proton and abstraction of the C-α-proton [29]. Investigation of a *Pseudomonas putida* MGL mutant form with substitution of active site tyrosine 114 with phenylalanine showed that Tyr114 most likely acted as a general acid catalyst at the stage of leaving group elimination [50].

In [56], the authors analyzed the spatial structures of four intermediates formed during the interaction of* Entamoeba hystolytica *MGL with L-methionine and proposed a γ-elimination reaction mechanism different from the above scheme, which excluded the stages of two quinonoid intermediates (*Scheme*, intermediates 3 and 9). This mechanism assumes the formation of α-aminocrotonate following β,γ-unsaturated ketimine due to a 1,5-sigmatropic proton shift from the C4’- atom to the Cγ-atom, which does not require catalysis.

**Scheme:**
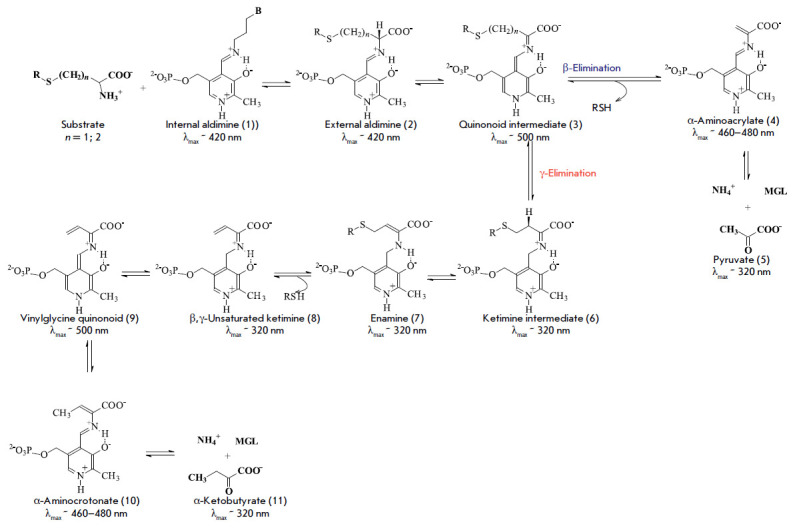
Mechanism of β- and γ-elimination reactions catalyzed by PLP-dependent enzymes

**Steady-state kinetic parameters, inhibition of the γ-elimination reaction, and exchange of C-α- and C-β-protons in Ser339Ala MGL complexes with inhibitors**

*Table 2* shows the steady-state kinetic parameters of γ- and β-elimination reactions catalyzed by the wild-type MGL [35, 36, 57] and mutant form. Compared with the wild-type enzyme, substitution of Ser339 with Ala led to a decrease in the rates of β-elimination by 5- to 10-fold and γ-elimination by 30- to 40-fold. The KM values increased more for substrates containing a leaving group in the β-position, and, as a result, the catalytic efficiency for both reactions decreased equally by two orders of magnitude, on average. In the case of substrates with a good leaving group, the catalytic efficiency of the mutant form in the γ-elimination reaction is comparable to that of the wild-type MGL but the kcat/KM parameter is two orders of magnitude lower in the β-elimination reaction of O-Ac-L-Ser.

**Table 2:** Kinetic parameters of γ- and β-elimination reactions

Substrate	MGL, wild type			MGL, Ser339Ala		
	k_cat_, s^–1^	K_M_, mM	k_cat_/K_M_, M^–1s^^–1^	k_cat_, s^–1^	K_M_, mM	k_cat_/K_M_, M^–1s^^–1^
L-Met	6.2 ± 0.42*	0.7 ± 0.11*	8.85 × 10^3^	0.21 ± 0.002	1.84 ± 0.15	1.77 × 10^2^
DL-Hcy	8.51 ± 0.41*	0.97 ± 0.15*	8.77 × 10^3^	0.28 ± 0.009	3.39 ± 0.34	8.25 × 10^1^
S-Et-L-Hcy	6.78 ± 0.02*	0.54 ± 0.01*	1.25 × 10^4^	0.16 ± 0.0016	0.54 ± 0.037	2.92 × 10^2^
O-Ac-L-Hse	2.1 ± 0.053**	2.91 ± 0.18**	7.21 × 10^2^	0.77 ± 0.011	2.07 ± 0.22	3.73 × 10^2^
S-Met-L-Cys	4.6 ± 0.29*	0.71 ± 0.11*	6.48 × 10^3^	0.41 ± 0.018	21.8 ± 2.25	1.88 × 10^1^
S-Et-L-Cys	5.03 ± 0.16*	0.17 ± 0.02*	2.96 × 10^4^	0.67 ± 0.024	6.47 ± 0.54	1.04 × 10^2^
S-Bzl-L-Cys	8.16 ± 0.23*	0.18 ± 0.02*	4.53 × 10^4^	1.81 ± 0.094	5.76 ± 0.59	3.14 × 10^2^
O-Ac-L-Ser	2.13 ± 0.037***	4.28 ± 0.33***	4.98 × 10^2^	0.047 ± 0.001	16.36 ± 1.09	2.87

^*^Data from [35].

^**^Data from [36].

^***^Data from [57].

Glycine, L-alanine, L-α-aminobutyric acid, L-norvaline, and L-norleucine are competitive inhibitors of the physiological reaction for both the mutant enzyme and the wild-type enzyme. The inhibition constants were found to be comparable to those of the wild-type MGL (*Table 3*).

**Table 3:** Inhibition of the γ-elimination reaction of L-methionine and the kinetic parameters of isotopic exchange of the C-α- and C-β-protons in inhibitors

Amino acid	MGL, wild type			MGL, Ser339Ala			Number of exchanged K C-α- and C-β-protons
	*K*_I_, mM	k_ex_, s^-1^ K_M_ = K_P_, mM		*K*_I_, mM	k_ex_, s^-1^ K_M_ = K_P_, mM		
		α-H	β-H		α-H	β-H	
Gly	48.49 ± 4.37*	20.2*	–	22.87 ± 2.84	0.078	–	1, pro-(R) proton**
L-Ala	3.41 ± 0.40*	2.71*	2.63*	1.25 ± 0.32	0.387	0.116	1; 3
L-α-Abu	8.01 ± 0.76*	–	–	4.66 ± 0.51	–	–	
L-Nva	4.60 ± 0.43*	–	–	1.7 ± 0.32	–	–	
L-Nle	0.6 ± 0.06*	41.8*	4.74*	0.89 ± 0.09	0.46	0.12	1; 2

^#^KI is the inhibition constant; KM is the Michaelis constant; KP is the product inhibition constant which characterizes the binding of the enzyme to the product of the isotope exchange.

^*^Data from [36].

^**^Data from [40].

The data of isotopic exchange of substrate and inhibitor protons enable an assessment of the contribution of individual stages to the enzymatic reaction and the elucidation of the stereochemistry of the proton exchange. *Table 3* summarizes the rates of C-α- and C-β-proton exchange in the inhibitors, which was catalyzed by Ser339Ala MGL, compared to those of the wild-type MGL. The substitution led to a slowdown in the rates of C-α- and C-β-proton exchange in the inhibitors. A decrease in the exchange rate of the C-α-proton in the complexes of mutant enzyme with glycine and norleucine compared with those of the wild-type enzyme was two orders of magnitude; the exchange rates of C-β-protons decreased by an order of magnitude.

An investigation of the stereospecificity of the glycine proton exchange in the wild-type enzyme showed that the ratio of exchange rates of the *pro*-(R) and* pro*-(S) protons was 14,000 : 1 [40]. The 1H NMR spectrum of a dipeptide L-phenylalanylglycine (data not shown) containing glycine, which was isolated after incubation of the mutant enzyme with glycine in D2O, had a signal at 3.4 ppm characteristic of the methylene* pro*-(R) proton of glycine [41].

We were unable to detect any exchange of the *pro*- (S) proton after incubation of the mutant form with glycine for a long period of time, because it led to the inactivation of Ser339Ala MGL. The obtained data showed that both the wild-type enzyme and the mutant MGL predominantly exchanged the *pro*-(R) proton.

**Spectral studies**

**Fig. 1:**
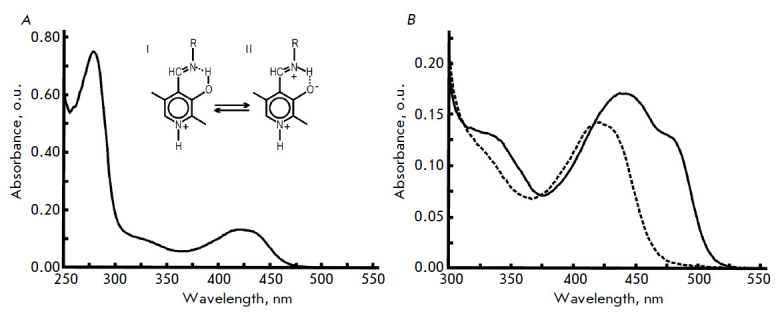
Absorption spectra: (*A*) Ser339Ala MGL holoenzyme; (*B*) MGL–L-methionine complexes: wild-type MGL (solid line), and Ser339Ala MGL (dashed line). The spectra were acquired in a 50-mM potassium phosphate buffer, pH 8.0, containing 1 mM DTT, 1 mM EDTA, and 1 mg/mL of the enzyme

*Figure 1* shows the absorption spectra of the Ser339Ala holoenzyme and its complex with L-methionine. The absorption spectrum of the mutant enzyme has a form typical of PLP aldimines, with the main band at 420 nm and a band at 320 nm.

The results of lognormal deconvolution of the holoenzyme spectrum, which was carried out as described for the wild-type enzyme [37], are shown in *Table 4*. The internal aldimine of the mutant form is described by four structures: ketoenamine, its tautomer, enolimine (*Fig. 1A*; structures II and I), and two ketoenamine conformers, a conformer whose aldimine group is in a plane perpendicular to the pyridine ring plane (*Table 4*, structure II┴), and a conformer whose aldimine bond is out of the coenzyme ring plane but retains conjugation with π-electrons of the coenzyme ring and a hydrogen bond between the aldimine nitrogen atom and the coenzyme 3’-hydroxy group (*Table 4*, structure II┴). The absorption band parameters obtained from spectral deconvolution are summarized in *Table 4*.

**Table 4:** Parameters of the absorption spectrum of the Ser339Ala MGL internal aldimine

Structure	E, eV	ν × 10^–3^, cm^–1^	λ, nm	ε × 10^–3^, M–1cm^–1^	W × 10^-3^, cm^–1^	ρ	f	n, %
II^1^	2.92	23.58	424.1	10.46	3.58	1.55	0.18	52.9
II^∠^	3.24	26.10	383.1	8.27	3.87	1.37	0.03	10.4
I	3.63	29.26	341.8	9.75	3.65	1.23	0.06	20.0
II^┴^	3.80	30.67	326.1	8.50	3.47	1.29	0.05	16.7
II^2*^	4.28	34.52	289.7	12.20	5.06	1.20	0.29	
*	4.55	36.56	272.8	23.10	4.56	1.39	0.79	

E is the electronic transition energy; ν is the wave number; λ is the wavelength; ε is the molar absorption coefficient; W is the half-width; ρ is the asymmetry; f is the oscillator strength; n is the content of tautomers and conformers.

The PLP content in the enzyme is 97%.

^*^Experimental information on these bands is insufficient. Superscripts (1, 2) refer to the first and second electronic transitions in structure II. Superscripts (┴ and ∠) refer to two conformers of structure II (a conformer with an aldimine bond located in a plane perpendicular to the pyridine ring plane, and a conformer with an aldimine bond that is partially out of the pyridine ring plane but retains conjugation with the π-electrons of the cofactor ring and the hydrogen bond between the aldimine nitrogen and the 3’-oxy group of the coenzyme).

The discovered structures and the parameters of their absorption bands turned out to be almost identical to those of the wild-type enzyme [36]. The dissociation constant of the PLP complex for the wild-type MGL was 6.24 × 10^–4^ mM [57]. The PLP dissociation constant for the mutant enzyme was 1.01 × 10^–3^ mM. Thus, the substitution did not significantly affect the apoenzyme affinity for the coenzyme and the conformation and tautomerism of the internal aldimine. The substitution led to some change in the quantitative composition of the tautomers and conformers of the internal aldimine. The reactive form of internal aldimine is ketoenamine. Its content is 67.6% in the wildtype holoenzyme [36] and 52.9% in the mutant holoenzyme (*Table 4*).

Absorption and the circular dichroism spectra of the wild-type MGL complexed with L-methionine display bands with maxima at 440 nm and 480 nm [36]. These bands in the spectra of PLP-dependent enzymes are assigned to β- and γ-elimination reaction intermediates: α-aminocrotonate or α-aminoacrylate [54]. The presence of α-aminocrotonate in the spectrum of the wild-type MGL associated with L-methionine and the kinetic data on the interaction between the wild-type enzyme and a number of substrates and inhibitors suggest that the rate-limiting stage of the physiological reaction follows the stage of α-aminocrotonate formation [36]. There is no absorption at 440–480 nm in the absorption spectrum of the mutant enzyme associated with L-methionine. Consequently, the substitution of Ser339 with Ala led to the inhibition of the γ-elimination reaction at the stage(s) following the stage of the external aldimine.

**Spatial structure of the mutant holoenzyme**

**Fig. 2:**
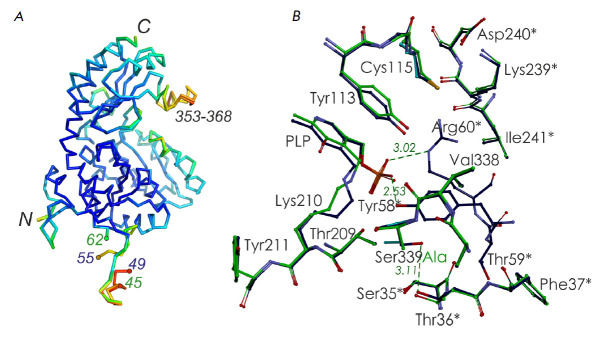
Superposition of the structures of the wildtype* C. freundii *MGL (code PDB 2RFV; green) and Ser339Ala MGL (code PDB 5D5S; dark blue): (*A*) the polypeptide chain is colored according to the B-factor, varying from dark blue to red as the value increases; (*B*) fragments of active centers; asterisks indicate residues belonging to the neighboring subunit of the catalytic dimer

The spatial structure of Ser339Ala MGL was determined at a resolution of 1.7 Å. The substitution of serine 339 with alanine did not affect the spatial structure of the enzyme. The course of the polypeptide chain was almost identical to that of the wild-type MGL holoenzyme (*Fig. 2A*). The root-mean-square deviation of the Cα-atom positions of Ser339Ala MGL compared to their positions in the wild-type enzyme (PDB code 2RFV) is 0.29 Å2. As in the previously determined structures of *C. freundii *MGL [29, 30, 31] and the structures of MGL from other microorganisms, the Ser339Ala MGL structure is characterized by a flexibility of the N- and C-terminal fragments of the polypeptide chain (*Fig. 2A*) [28]. The high flexibility of the N-terminal fragment in Ser339Ala MGL precluded the localization of a long segment consisting of sixteen amino acids (46–61), including Tyr58 and Arg60, whose side groups form hydrogen bonds with the O_2_P atom of the phosphate “handle” of the coenzyme (*Fig. 2B*).

The average temperature B-factor of the amino acid residues from the flexible C-terminal fragment (54.02 Å2, residues 353–368) is two-fold higher than that of the stable regions of the enzyme. This flexibility may be associated with removal of γ- and β-elimination products from the active center [30]. The substitution of Ser339 with alanine led to the loss of the hydrogen bond between it and Thr36 of the neighboring subunit of the catalytic dimer (*Fig. 2B*), but this did not affect the tetrameric structure of Ser339Ala MGL.

**Spatial structures of the covalent and noncovalent complexes of MGL with inhibitors and the role of Ser339 in the catalysis of the γ- and β-elimination reactions**

**Fig. 3:**
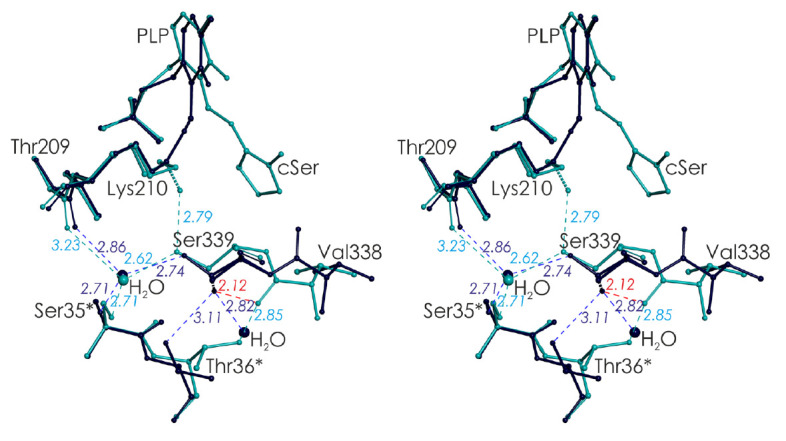
Stereoview of the superposition of active center fragments of* C. freundii *MGL: the wild-type holoenzyme (PDB code 2RFV, dark blue) and its complex with L-cycloserine (PDB code 4OMA, blue). Asterisks indicate residues belonging to the neighboring subunit of the catalytic dimer. The alternative positions of the amino acid residues are shown by dashed lines

In the spatial structure of the *C. freundii *wild-type MGL holoenzyme (PDB code 2RFV), the Ser339 side chain occurs in two equally probable positions (*Fig. 3*). In one of them, it is located in the active site, next to Lys210; in the second position, it is located in the region of intersubunit interaction with the N-terminal domain of the neighboring subunit of the catalytic dimer. Both positions of the Ser339 side chain are stabilized by hydrogen bonds and are clearly visible on the electron density map.

In the spatial structures of MGL complexes with inhibitors, which model the Michaelis complex, external aldimine, and the ketimine intermediate, the Ser339 side chain occupies the only position in which its Oγ-atom is directed towards Lys210 [29, 30, 31, 32]. When MGL binds inhibitors, their carboxyl groups push the carbonyl group of the peptide bond between Ser339 and Val338 from the active center cavity into the region of intersubunit contacts and the C=O-group is rotated by 180° [29, 32]. Upon this rotation, the distance between the Oγ-atom of Ser339 and the oxygen atom of the Val338 main chain drops to 2.12 Å, which leads to the thrusting of the Oγ-atom of Ser339 into the enzyme active site region (*Fig. 3*). This ensures the only position of the Ser339 side chain in which its Oγ-atom forms a hydrogen bond with the Nζ-atom of Lys210. The bond length is 2.85 Å in the Cys115His MGL–L-norleucine complex modeling the external aldimine [31] and 2.79 Å in the wild-type MGL–Lcycloserine complex modeling the ketimine intermediate [30] (*Fig. 3*).

As noted above, the MGL N-terminal fragment is flexible both in the wild-type holoenzyme and in the Ser339Ala MGL holoenzyme. Binding of inhibitors by wild-type MGL leads to a stabilization of this *A B* fragment [30, 32]. Probably, this stabilization also occurs upon binding of substrates and inhibitors by Ser339Ala MGL.

**Fig. 4:**
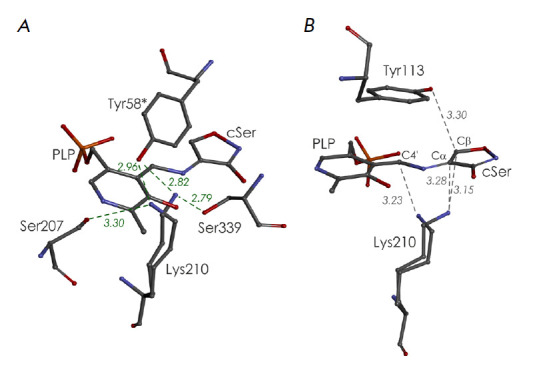
(*A*) Environment of Lys210 in the active center of the *C. freundii *MGL–L-cycloserine complex (PDB code 4OMA). Asterisks indicate amino acid residues belonging to the second subunit of the catalytic dimer. (*B*) Position of the Nζ-atom of the Lys210 side chain relative to the C4’-atom of PLP and Cα- and Cβ-atoms of the inhibitor

*Figure 4* shows the active center fragments of the* C. freundii *MGL–L-cycloserine complex, which represent two positions of the Lys210 side chain.

In one position, which is analogous to the Lys210 side chain position in the mutant form–norleucine complex [31] modeling an external aldimine, the Lys210 Nζ-atom is stabilized by hydrogen bonds with the hydroxyl groups of the Ser339 and Tyr58* side chains (2.79 Å and 2.82 Å, respectively; *Fig. 4A*). The side chain of Lys210 is almost perpendicular to the coenzyme ring plane and occurs at a distance of 3.28 Å from the C-α-*pro*-(R)-proton and 3.15 Å from the Cβ-atom of L-cycloserine (*Fig. 4B*). This position is favorable to the abstraction of the C-α-*pro*-(R)-proton or C-β-proton by the side amino group of Lys210.

In another position, the Nζ-atom of Lys210 is located at a hydrogen bond distance from the hydroxyl groups of the Ser207 and Tyr58* side chains (3.30 Å and 2.96 Å, respectively; *Fig. 4A*). In this position, the Nζ-atom of Lys210 is closest (3.23 Å) to the C4’-atom of PLP, while the distance to the Cα-atom of the substrate increases by 0.43 Å (*Fig. 4B*). This position of the Lys210 side chain is favorable to a transfer of the C-α-proton to the C4’-atom of the coenzyme.

Therefore, if Tyr58* is involved in the stabilization of both positions of the Lys210 Nζ-atom, then the Ser339 and Ser207 side chains, located on opposite sides of Lys210, provide the optimal position for the Lys210 ε-amino group at the stages of C-α-proton abstraction in the external aldimine, protonation of the C4’-atom of PLP, and abstraction of the C-β-proton in the ketimine intermediate.

In the mutant form complexed with substrates and inhibitors, the Lys210 group can form only one hydrogen bond with the Tyr58* hydroxyl group. This leads to a disruption of the Lys210 side chain conformation optimal for the abstraction of the C-α-proton and to a decrease in its pKa. Obviously, this explains the decrease in the observed exchange rate of the C-α- proton of inhibitors. The exchange of C-β-protons was previously thought to occur via the configuration inversion mechanism when the first proton is abstracted by the Lys210 side chain, and the second proton comes from the opposite side: from the Tyr113 side chain. As a result, both C-β-protons are exchanged at the same rate [29]. In the Ser339Ala enzyme, the rate of isotopic exchange of C-β-protons decreases and both β-protons undergo exchange at the same rate.

Probably, the hydrogen bond between the Nζ-atom of Lys210 and the Oγ-atom of Ser339 ensures both a Lys210 amino group position optimal for abstraction of the C-α- and C-β-protons, and stabilization of its ammonium form for the 1,3-prototropic shift of the C-α-proton to the C4’-atom of the coenzyme and effective exchange of C-α- and C-β-protons in complexes with substrates and inhibitors. The absorption band of an external aldimine in the absorption spectrum of the Ser339Ala–L-methionine complex also suggests that the substitution of Ser339 with alanine leads to inhibition of the physiological reaction at the stage of C-α-proton abstraction from the external aldimine.

If α-aminocrotonate formation occurs via the mechanism proposed in [50], the ε-amino group of Lys210 can participate as a base or an acid in the physiological reaction stages that follow the elimination of methyl mercaptan and the substitution of Ser339 can slow these stages of the physiological reaction.

In the β-elimination reaction, the substitution of serine 339 with alanine leads to inhibition at the stage of abstraction of the C-α-proton of the external aldimine. Because Lys210 is postulated as a general acid catalyst in PLP-dependent β-elimination reactions of sulfur-containing amino acids [54], the hydroxyl group of Ser339 probably provides a position and basicity of Lys210 that are optimal for catalysis.

Earlier, a similar role for a serine residue corresponding to MGL Ser339 was proposed for two enzymes from the structural subclass to which MGL belongs: cystathionine γ-synthase [58] and cystathionine β-lyase [59].

## CONCLUSION

In this study, we have demonstrated the importance of the Ser339 residue in the mechanism of γ- and β-elimination reactions catalyzed by *C. freundii *MGL. Kinetic, spectral, and X-ray data revealed that Ser339 is necessary to ensure an optimal position of the Lys210 side chain at the stage of C-α-proton abstraction from the substrate in the β-elimination reaction and at the stages of C-α- and C-β-proton abstraction from the substrate in the γ-elimination reaction. In the γ-elimination reaction, the Ser339 residue is supposed to ensure the necessary basicity of the Lys210 side chain at the stage of the 1,3-prototropic shift of the C-α-proton of the substrate to the C4’-atom of the coenzyme. Along with the contribution to basic enzymology, understanding the mechanisms of γ- and β-elimination reactions catalyzed by MGL is necessary for the development of new antibacterial and antitumor drugs based on changing/improving the substrate and reaction specificity of the enzyme.

## Abbreviations

MGL: methionine γ-lyase
PLP: pyridoxal 5’-phosphate
Ser339Ala: a mutant form of the enzyme with Ser339 replaced by Ala
LDH: lactate dehydrogenase
HOHxoDH: D-2-hydroxyisocaproate dehydrogenase
NADH: reduced nicotinamide adenine dinucleotide
